# Measuring burden of disease in both asthma and COPD by merging the ACQ and CCQ: less is more?

**DOI:** 10.1038/s41533-024-00364-z

**Published:** 2024-05-03

**Authors:** Liz J. A. Cuperus, Cathelijne M. van Zelst, Huib A. M. Kerstjens, Rudi W. Hendriks, Maureen P. M. H. Rutten-van Molken, Jacqueline B. Muilwijk-Kroes, Gert-Jan Braunstahl, Johannes C. C. M. in ’t Veen

**Affiliations:** 1 Pulmonology Department, Franciscus Gasthuis and Vlietland, Rotterdam, the Netherlands; 2 Pulmonology Department, Erasmus Medical Center, Rotterdam, the Netherlands; 3 Pulmonology Department, University of Groningen, University Medical Center Groningen, Groningen, the Netherlands; 4 Erasmus School of Health Policy & Management, Erasmus University, Rotterdam, The Netherlands; 5 Star-Shl, Diagnostic Center Primary Care, Rotterdam, the Netherlands

**Keywords:** Chronic obstructive pulmonary disease, Asthma, Quality of life, Medical research

## Abstract

Symptoms of asthma and COPD often overlap, and both diseases can co-exist in one patient. The asthma control questionnaire (ACQ) and clinical COPD questionnaire (CCQ) were developed to assess disease burden in respectively asthma or COPD. This study explores the possibility of creating a new questionnaire to assess disease burden in all obstructive lung diseases by integrating and reducing questions of the ACQ and CCQ. Data of patients with asthma, COPD and asthma-COPD overlap (ACO) were collected from a primary and secondary care center. Patients completed ACQ and CCQ on the same day. Linear regression tested correlations. Principal Component Analysis (PCA) was used for item reduction. The secondary cohort with asthma and COPD patients was used for initial question selection (development cohort). These results were reproduced in the primary care cohort and secondary cohort of patients with ACO. The development cohort comprised 252 patients with asthma and 96 with COPD. Correlation between ACQ and CCQ in asthma was R = 0.82, and in COPD R = 0.83. PCA determined a selection of 9 questions. Reproduction in primary care data (asthma *n* = 1110, COPD *n* = 1041, ACO = 355) and secondary care data of ACO patients (*n* = 53) resulted in similar correlations and PCA-derived selection of questions. In conclusion, PCA determined a selection of nine questions of the ACQ and CCQ: working title ‘the Obstructive Lung Disease Questionnaire’. These results suggest that this pragmatic set of questions might be sufficient to assess disease burden in obstructive lung disease in both primary as secondary care.

## Introduction

Chronic obstructive pulmonary disease (COPD) and asthma are both obstructive pulmonary diseases with a high disease burden. Although the diagnoses of COPD and asthma are clearly defined, the symptoms and clinical manifestations of COPD and asthma show considerable overlap, e.g., wheeze, dyspnea, and cough. Additionally, some patients seem to have both diseases: the Global Initiative for Chronic Obstructive Lung Disease (GOLD) and the Global Initiative for Asthma (GINA) previously described this condition as asthma-COPD overlap (ACO)^1^. In today’s context, there exists some controversy surrounding this term, with the current terminology often describing this group as COPD patients with asthma features or asthma patients with COPD features. Nevertheless, some studies suggest that the prevalence of ACO can be as high as 25% in patients with COPD and 31% in adult patients with asthma; this means that a substantial part of the patients with COPD or asthma have features of both diseases^2,3^. Furthermore, the prevalence of ACO increases with age, so it might be expected that this group will be even larger in the future due to the aging population^4^.

The asthma control questionnaire (ACQ) and clinical COPD questionnaire (CCQ) were developed to assess the disease burden in patients with respectively asthma or COPD^5,6^. The ACQ and CCQ are short, practical, and are used regularly in primary, secondary, and tertiary care in the Netherlands^7,8^. Because of the similarities in clinical features of asthma and COPD, the diagnosis may not be clear in the beginning, and the diagnostic process can take several consultations. During this initial period, both the ACQ and the CCQ have to be completed to assess disease burden, resulting in extra work for patients and health care professionals^9,10^. Moreover, patients with features of both diseases were excluded from the development and validation of the ACQ and CCQ, so these questionnaires may be less appropriate for patients with ACO^5,6^. A single, practical questionnaire for both diseases is needed to improve the assessment of disease burden in this substantial proportion of the patients with asthma and COPD, which could also be useful for those with asthma or COPD alone.

The aim of our study was to explore the possibility of creating a single questionnaire for assessment of the disease burden in asthma, COPD, and ACO. We hypothesize that this new approach, containing a selection of questions from the ACQ and CCQ, could be used to assess disease burden and quality of life in asthma, COPD and ACO.

## Methods

### Study design

In this study, retrospective cross-sectional cohort analyses were performed in patients with asthma and COPD treated in a secondary care cohort. In the first analysis, we aimed to compare two disease burden questionnaires and tested the correlation between the questionnaires. Patients completed both the ACQ and the CCQ on the same day, and these were compared separately in the asthma group and COPD group. Second, we selected questions of the ACQ and CCQ based on data reduction of the two questionnaires in a development cohort. Thirdly, we reproduced this selection process by data reduction in three reproduction cohorts: (1) a secondary cohort of patients with ACO; (2) a primary care cohort with asthma or COPD; and (3) a primary care cohort with ACO patients. Patients with ACO in the secondary cohort were not included in the development cohort, because this was a small patient group. The questions for this potential new questionnaire were selected by comparing and combining the results of the development cohort and the results of the three reproduction cohorts. Finally, we took the first step in testing the validity and reliability of this new potential questionnaire.

### Setting and participants

The development cohort comprises patients with asthma and COPD in secondary care. Data were part of a registry study of adult patients with asthma or COPD. Patients were newly referred to the Franciscus Gasthuis and Vlietland in Rotterdam, the Netherlands, a center of excellence for asthma and COPD care. The diagnosis was confirmed by a pulmonologist with a special interest in obstructive lung disease during a previously published comprehensive assessment^11^. Patients were included between December 2012 and December 2017. Both ACQ and CCQ had to be completed on the same day. Diagnostic criteria of the GINA and GOLD report were used for respectively asthma and COPD. Asthma diagnosis was based on the presence of typical clinical symptoms, reversible airway obstruction ( + 12% and 200 ml improvement in FEV_1_ after bronchodilator) or bronchial hyperreactivity (PC20 < 8 mg/ml) or a FeNO > 50 ppb^12^. The diagnosis of COPD was based on a clinical assessment (e.g. medical history, exposure, and age) by a pulmonologist in combination with spirometry (post-bronchodilator forced expiratory volume (FEV_1_) / forced vital capacity (FVC) < 0.7)^13^. ACO was defined by using the definition of the joint report of the GINA and GOLD in 2015: persisted airflow limitation with features of both asthma and COPD^1^. In this study, we used pseudonymized data. Ethics approval for this study was waived by the Institutional Research Board of the Franciscus Gasthuis & Vlietland, Rotterdam, the Netherlands (identification number 2017-084,) because routinely collected health care data were used after pseudonymization.

The process of selecting questions from the ACQ and CCQ to form the new questionnaire was reproduced in three separate reproduction cohorts: (1) ACO group in secondary care dataset; (2) the asthma and COPD group from the primary care dataset; (3) ACO group from the primary care dataset. Patients who exhibited characteristics of both asthma and COPD, as determined by the pulmonologist during assessment at the secondary care center, were enrolled in the first reproduction cohort. Data of the second and third cohort were part of a registry study of patients with asthma, COPD or ACO, who were diagnosed by Star-Shl, a diagnostic center for primary care in Rotterdam. Diagnoses of COPD and asthma were confirmed by spirometry and by a general practitioner or pulmonologist with special interest in obstructive lung diseases, using the same criteria as the development cohort^12–14^. ACO patients were analyzed separately because this patient group was smaller and not clearly defined compared to the asthma and COPD groups. Data were pseudonymized and ethics approval for this study was waived by Star-Shl in line with the waiver procedure for the secondary care cohort.

### Data collection

The following variables were collected for all patients:

#### Lung function

FEV_1_ and FER (FEV_1_/FVC) were performed according to the ATS/ ERS taskforce “standardization of spirometry”. All tests in the secondary cohort were performed with the Vmax Sensor Medics Viasys, type 6200 Encore^15^. In the primary cohort (Star-Shl), all spirometry studies were performed with the Welch Allyn Cardioperfect spirometer.

#### Clinical COPD questionnaire (CCQ)

This is a ten-item questionnaire about symptom severity in the past seven days and health-related quality of life. CCQ total score ranges from 0 to 6, where a higher score indicates a worse health status^6^. The minimal clinically important difference of the CCQ is 0.4^16^. CCQ-1 is a question about the shortness of breath at rest and CCQ-2 is about shortness of breath during physical activities. CCQ-3 is about concerns of getting a cold or breathing getting worse, CCQ-4 is about depressive feelings due to breathing problems, CCQ-5 is about coughing, and CCQ-6 is about the production of phlegm. The four last questions are about limitations during activities because of breathing problems: limitations during strenuous physical activities (CCQ-7), moderate physical activities (CCQ-8), daily activities at home (CCQ-9), or social activities (CCQ-10).

#### Asthma control questionnaire (ACQ)

This questionnaire assesses average symptom severity and control in asthma in the past week. ACQ total score ranges from 0 to 6, where a higher score indicates a worse disease control^5^. We used the five-item questionnaire, according to the preference of the GINA guideline^10^. ACQ-1 is a question about the frequency of wakening due to asthma symptoms, ACQ-2 is about the severity of symptoms during wakening, ACQ-3 is about limitation during activities, ACQ-4 is about the severity of shortness of breath, and ACQ-5 is about the frequency of wheezing. The minimal clinically important difference is 0.5^17^. The ACQ and CCQ share a comparable scoring system and partially similar format since the original developers collaborated in designing these questionnaires^5,6^.

To study the construct validity of the subset of questions that resulted from the Principal Component Analysis (PCA), we administered the *COPD Assessment Test (CAT)* in patients with COPD and the *Asthma Quality of Life Questionnaire (AQLQ)* in patients with asthma. A higher score in the eight-item questionnaire CAT reflects a worse outcome and in the 32-item questionnaire AQLQ a lower score reflects more impairment^18,19^.

### Statistical analyses

At first, linear regression was used to test for correlation between the ACQ and CCQ. The Pearson correlation coefficient (R) was calculated separately for the asthma, COPD, and ACO patients in both the secondary and primary care cohort. The R assesses the strength and direction of the linear relationship between two variables. Values range from -1 to 1 with values close to zero meaning a weak linear relationship and those approaching -1 or 1 signifying a strong negative or positive relationship, respectively.

Secondly, Principal Component Analysis (PCA) was used to identify the questions of the ACQ and CCQ that could be used to develop the new questionnaire. Based on the correlation between the individual questions, PCA reduced the number of questions by replacing them with newly created variables (‘components’) with minimal information loss. This method was used to identify trends in the answers to the questionnaires and understand what these answers have in common. To develop a component an eigenvalue > 1 was used. Oblimin with rotation, converged in 25 iterations, was used with method Kaiser Normalization. The component included questions that met correlation cut-off values of 0.7 and -0.7. The question with the highest correlation in the component was chosen as its identifier. To ensure there was no cross-talking between components, the identifying question was examined to see if any other variable had a loading of more than 0.4 on the same component. If this occurred, the question could not be used as the component’s identifier^20^. Questions that were not related to any of the components were added individually to the final questionnaire to ensure that valuable information from those questions was not lost. Alpha Cronbach’s was used to measure internal consistency of the new questionnaire. The Cronbach’s alpha coefficient ranges from 0 to 1 and refers to the degree to which all the items within a test assess the same underlying concept. Values close to 1 indicate high internal consistency; values closer to 0 suggest low internal consistency. Third, three reproduction cohorts were used to repeat this process of selecting the questions by PCA. The same conditions of the PCA were investigated: Eigenvalue > 1, cut-off values of 0.7 and Oblimin with rotation, converged in 25 iterations, with method Kaiser Normalization.

Fourth and final, first steps were initiated to test the validity and reliability of this prospective new questionnaire. New correlation plots were performed in asthma and COPD separately to test the correlation between the new questionnaire and the golden standard: the ACQ in patients with asthma and the CCQ in patients with COPD. Furthermore, we calculated a Pearson correlation coefficient test to study the construct validity of the new selection of questions with the AQLQ in asthma and CAT in COPD. All statistical analyses were performed using IBM SPSS Statistics, version 28.0.0.0 (190).

### Reporting summary

Further information on research design is available in the Nature Research Reporting Summary linked to this article.

## Results

### Patient characteristics

**Fig. 1:**
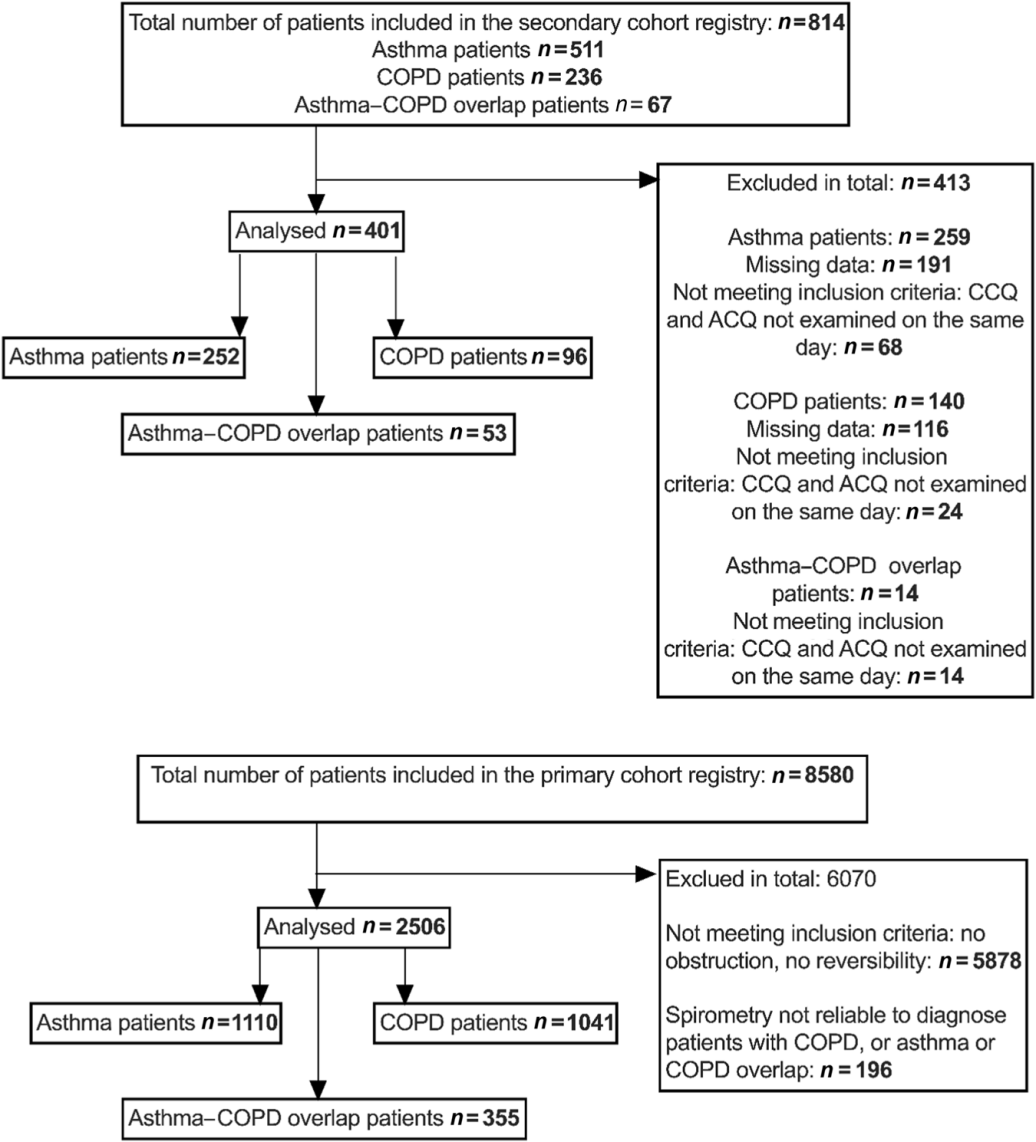
Patient enrolment of secondary care cohort and primary care cohort.

In total 814 patients were considered in the development cohort (secondary care cohort). Patients were excluded for this study because of missing data (*n* = 307) or when CCQ and ACQ were not completed on the same day (*n* = 106). So, in total 252 asthma patients and 97 COPD patients were included in the development cohort, Fig. 1. In the asthma group 162 (64.3%) were female and in the COPD group 42 (43.8%), Table 1. The median age [IQR] of the asthma patients was 48.5 years old [38.3–59.0] and in COPD 63.0 years old [55.0–70.0]. There were more active smokers in the COPD group, compared to the asthma group (52.1% vs. 12.7%, <0.001). The median FEV-1 post bronchodilator percentage predicted [IQR] was higher in asthma patients compared to the COPD patients (93% [79–104] vs. 66% [52–82]). The items of the ACQ and CCQ differed significantly between asthma and COPD in the questions ACQ 1, ACQ 2, CCQ 1, and CCQ 6, Suppl. Fig. 1. Median scores of ACQ 2 and CCQ 1 were higher in asthma patients, compared to COPD patients and ACQ 1 and CCQ 6 scores were elevated in COPD patients.

**Table 1:** Patient characteristics in secondary and primary cohort.

	Secondary care cohort (*n* = 348)		Primary care cohort (*n* = 2151)	
Characteristics	Asthma (*n* = 252)	COPD (*n* = 96)	Asthma (*n* = 1110)	COPD (*n* = 1041)
Female sex, n(%)	162 (64.3)	42 (43.8)	629 (56.7)	496 (47.6)
Age, median [IQR]	48.5 [38.3-59.0]	63.0 [55.0-70.0]	43 [28–56]	64 [57–71]
BMI, median [IQR]	28.0 [24.4-32.4]	26.6 [21.6-29.5]	26.8 [23.5-30.6]	26.2 [23.5-29.4]
Active smokers, n(%)	32 (12.7)	50 (52.1)	223 (20)	510 (49)
FEV-1 pre percentage predicted, median [IQR]	83 [69–95]	62 [50–75]	79 [69–90]	67 [52–81]
FEV-1 post percentage predicted, median [IQR]	93 [79-104]	66 [52–82]	91 [82-101]	71 [56–84]
FEV-1/FVC, median [IQR]	75 [68–82]	54 [42–65]	76 [71–82]	58 [48–65]

*BMI* Body Mass Index in kg/m^2^. *FEV-1* Forced Expiratory Volume in 1 s.

The first reproduction cohort included 53 ACO patients from the secondary care data, Fig. 1. In this patient group 22 (41.5%) were female and 19 (35.8%) were active smokers. The median age was 61 [55–68], Suppl. Table 1. In total 1110 asthma patients and 1041 COPD patients of the primary care cohort were included in the second cohort, Fig. 1. In this primary care cohort, the patients in the COPD patient group were older (64 y [57–71] vs. 43 y [28–56]), and more men (52.4% vs. 44.3%) and active smokers were included compared to the asthma patient group (49% vs. 20%), Table 1. The third and final reproduction cohort included 355 ACO patients of the primary care dataset. In this group 165 (46.5%) were female and 148 (41.7%) were active smokers. The median age was 59 [50–68], mean FEV-1 post bronchodilator percentage predicted was 74.4 [61.4–84.6] and median FER was 60.7 [53.5–65.6], Suppl. Table 1.

### Correlation between the ACQ and CCQ

**Fig. 2:**
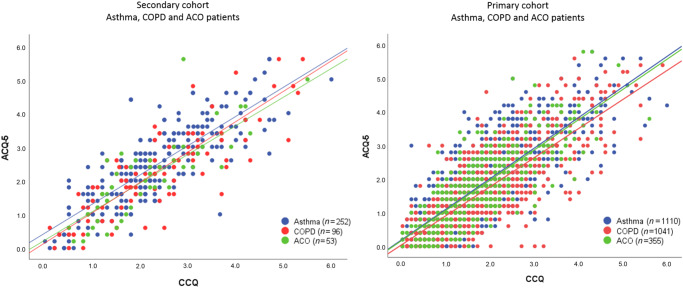
Correlation plot showing the correlation between the ACQ and CCQ in secondary cohort (left) and in primary cohort (right). In the secondary cohort, the Pearson correlation coefficient (R-score) was 0.82 in asthma patients, 0.83 in COPD patients, and 0.83 in ACO patients. In the primary cohort, the R-score was 0.81 in asthma patients, 0.80 in COPD patients and 0.81 in ACO patients.

In the secondary care cohort, the Pearson correlation coefficient (R) between the ACQ and CCQ was 0.82 in asthma patients, 0.83 in COPD patients, and 0.83 in ACO patients. In the primary care cohort, R was 0.81 in asthma patients, 0.80 in COPD patients and 0.81 in ACO patients, Fig. 2.

### Selecting questions of the ACQ and CCQ in the development cohort

**Fig. 3:**
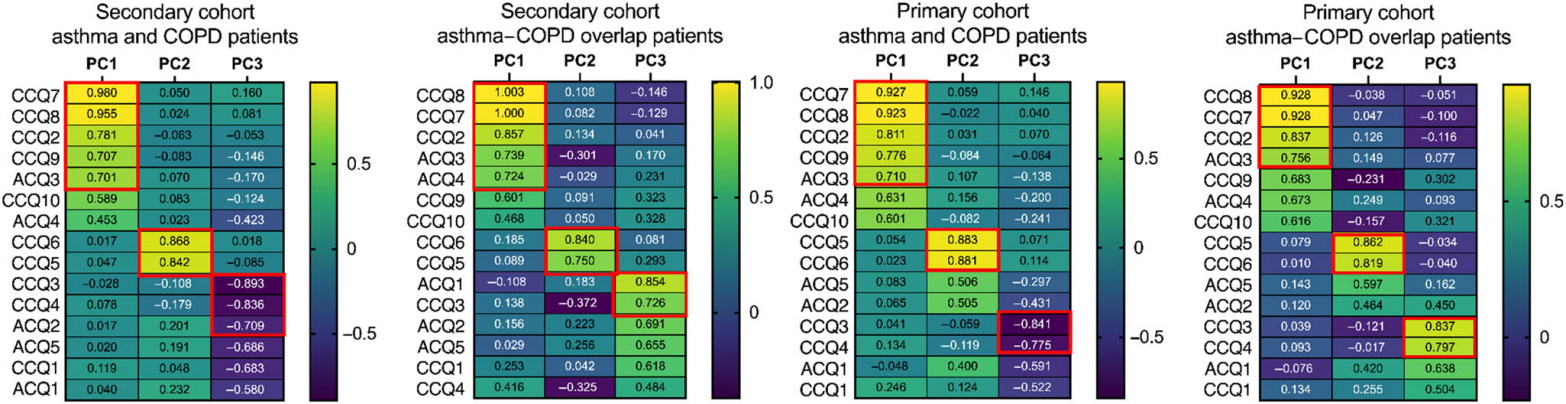
Results of Principal component analyses in secondary cohort and primary cohort, in asthma, COPD, and asthma-COPD overlap patients. The red box contains the questions of that particular component. PC1 = component 1; PC2 = component 2; PC3 = component 3. ACQ 1: on average, during the past week, how often were you woken by your obstructive lung disease during the night?; ACQ 2: on average, during the past week, how bad were your obstructive lung disease symptoms when you woke up in the morning?; ACQ 3: In general, during the past week, how limited were you in your activities because of your asthma?; ACQ 4: in general, during the past week, how much shortness of breath did you experience because of your obstructive lung disease?; ACQ 5: in general, during the past week, how much of the time did you wheeze?; CCQ 1: on average, during the past week, how often did you feel short of breath at rest?; CCQ 2: On average, during the past week, how often did you feel short of breath doing physical activities?; CCQ 3: On average, during the past week, how often did you feel concerned about getting a cold or your breathing getting worse?; CCQ 4: On average, during the past week, how often did you feel depressed (down) because of your breathing problems?; CCQ 5: In general, during the past week, how much of the time did you cough?; CCQ 6: in general, during the past week, how much of the time did you produce phlegm?; CCQ 7: on average, during the past week, how limited were you in these activities because of your breathing problems: strenuous physical activities (such as climbing stairs, hurrying, doing sports)?; CCQ 8: On average, during the past week, how limited were you in these activities because of your breathing problems: moderate physical activities?; CCQ 9: On average, during the past week, how limited were you in these activities because of your breathing problems: daily activities at home (such as dressing, washing)?; CCQ 10: on average, during the past week, how limited were you in these activities because of your breathing problems: social activities (such as talking, being with children, visiting friends/relatives).

Principal Component Analysis (PCA) was performed to integrate the ACQ and CCQ and reduce the number of questions. Three components were formed based on correlations between questions. Component 1 consists of: CCQ 7, CCQ 8, CCQ 2, CCQ 9, and ACQ 3. Component 2 consists of CCQ 6 and CCQ 5 and component 3 consists of CCQ 3, CCQ 4, ACQ 2. The CCQ 10, ACQ 4, ACQ 5, CCQ 1 and ACQ 1 were not correlated with the other items and therefore not included in a component, Fig. 3. This process of data reduction resulted in the selection of eight required questions: one question was selected for each component (CCQ 7 in component 1, CCQ 6 in component 2, CCQ 3 in component 3); and the residual questions (CCQ 10, ACQ 4, ACQ 5, CCQ 1, and ACQ 1).

### Reproduction of selection procedure in three cohorts

The selection procedure of the questions was repeated in three reproduction cohorts: (1) patients with ACO in the secondary cohort; (2) patients with asthma and COPD of in the primary care cohort; (3) the patients with ACO in the primary care cohort. As in the development cohort, the answers of ACQ and CCQ questions were combined into one dataset and questions were reduced by PCA.

The PCA of the data of the ACO patients in the secondary dataset showed similar results as the development cohort with a few exceptions. Component 1 also consists of CCQ 7, CCQ 8, CCQ 2, and ACQ 3; however, it contains the ACQ 4 instead of the CCQ 9. Component 2 in this reproduction cohort is identical to component 2 in the development cohort. Component 3 also consists of the CCQ 3, but contains the ACQ 1 instead of the CCQ 4 and ACQ 2, Fig. 3.

In the primary care data of the patients with asthma or COPD, the PCA resulted in similar components as in the development cohort; except for ACQ 2 which had a lower correlation value for component 2 in primary care in comparison with secondary care (0.505 vs. 0.709). Component 1 consists of resp.: CCQ 7, CCQ 8, CCQ 2, CCQ 9 and ACQ 3. Component 2 consists of CCQ 6 and CCQ 5 and component 3 consists of CCQ 3, CCQ 4. The residual questions were ACQ 1, ACQ 2. ACQ 4, ACQ 5, CCQ 1 and CCQ 10, Fig. 3.

The PCA with data of the asthma-COPD overlap patient group in the primary care data yielded similar results. Component 1 consists of resp.: CCQ 8, CCQ 7, CCQ 2, CCQ 9 and ACQ 3. Component 2 consists of CCQ 6 and CCQ 5 and Component 3 consists of CCQ 3, CCQ 4. The residual questions were ACQ 1, ACQ-2. ACQ 4, ACQ 5, CCQ 1 and CCQ 10, Fig. 3.

### Development of the Obstructive Lung Disease Questionnaire

Combining the results of the four PCA’s resulted in a selection of 9 questions. For each component, the question with the highest correlation in the development cohort was included in our selection. Component 1, containing the CCQ 7, CCQ 8, CCQ 2, CCQ 9 and ACQ 3, the CCQ-7 was selected as the identifying question of this component. These five questions were about complaints during physical activity; more specifically: shortness of breath during physical exercise (CCQ 2), limitations because of intense physical activity (CCQ 7), or moderate physical activity (CCQ 8), or daily activities (CCQ 9), or limitations because of activity (ACQ 3).

The CCQ 6 was selected from component 2. Both questions in component 2 are about coughing: CCQ 5 is about the amount of coughing and CCQ 6 is about the amount of sputum during coughing. CCQ 3 was selected as the identifying question of component 3, which contained the CCQ 3 and CCQ 4. CCQ 3 is a question about feeling concerned and the CCQ 4 about feeling depressed because of respiratory complaints. ACQ 2 was also statistically correlated with CCQ 3 and CCQ 4 in the development cohort. However, the ACQ 2 was not included in component 3 in any of the reproduction cohorts. Therefore, the ACQ 2 was not merged with the CCQ 3 and CCQ 4. The ACQ 1, ACQ 4, ACQ 5, CCQ 1 and CCQ 10 were not statistically correlated to any of the components. These questions were included in the final selection. This process resulted in a 9-item questionnaire with a 6 point scale with working title ‘’the Obstructive Lung Disease Questionnaire (OLD-Q)”. ‘’Asthma” was replaced for ‘’obstructive lung disease” to make the questionnaire applicable for all patients, Table 2.

**Table 2:** Working title ‘’Obstructive Lung Disease Questionnaire” as a potential new tool for measuring disease burden in obstructive lung disease.

1. On average, during the past week, how often were you woken by your obstructive lung disease during the night? *ACQ 1*	0 Never 1 Hardly ever 2 A few minutes 3 Several times 4 Many times 5 A great many times 6 Unable to sleep because of obstructive lung disease
2. On average, during the past week, how bad were your obstructive lung disease symptoms when you woke up in the morning? *ACQ 2*	0 No symptoms 1 Very mild symptoms 2 Mild symptoms 3 Moderate symptoms 4 Quite severe symptoms 5 Severe symptoms 6 Very severe symptom
3. In general, during the past week, how much shortness of breath did you experience because of your obstructive lung disease? *ACQ 4*	0 None 1 A very little 2 A little 3 A moderate amount 4 Quite a lot 5 A great deal 6 A very great deal
4. In general, during the past week, how much of the time did you wheeze? *ACQ 5*	0 Not at all 1 Hardly any of the time 2 A little of the time 3 A moderate amount of the time 4 A lot of the time 5 Most of the time 6 All the time
5. On average, during the past week, how often did you feel short of breath at rest? *CCQ 1*	0 Never 1 Hardly ever 2 A few times 3 Several times 4 Many times 5 A great many times 6 Almost all the time
6. On average, during the past week, how often did you feel concerned about getting a cold or your breathing getting worse? *CCQ 3*	0 Never 1 Hardly ever 2 A few times 3 Several times 4 Many times 5 A great many times 6 Almost all the time
7. In general, during the past week, how much of the time did you produce phlegm? *CCQ 6*	0 Never 1 Hardly ever 2 A few times 3 Several times 4 Many times 5 A great many times 6 Almost all the time
8. On average, during the past week, how limited were you in these activities because of your breathing problems: strenuous physical activities (such as climbing stairs, hurrying, doing sports)? *CCQ 7*	0. Not limited at all 1. Very slightly limited 2. Slightly limited 3. Moderately limited 4. Very limited 5. Extremely limited 6 Totally limited/ or unable to do
9. On average, during the past week, how limited were you in these activities because of your breathing problems: social activities (such as talking, being with children, visiting friends/relatives) *CCQ 10*	0. Not limited at all 1. Very slightly limited 2. Slightly limited 3. Moderately limited 4. Very limited 5. Extremely limited 6. Totally limited/ or unable to do

Circle the number of the response that best describes how you have been during the past week

### Validity and reliability of the Obstructive Lung Disease Questionnaire

**Fig. 4:**
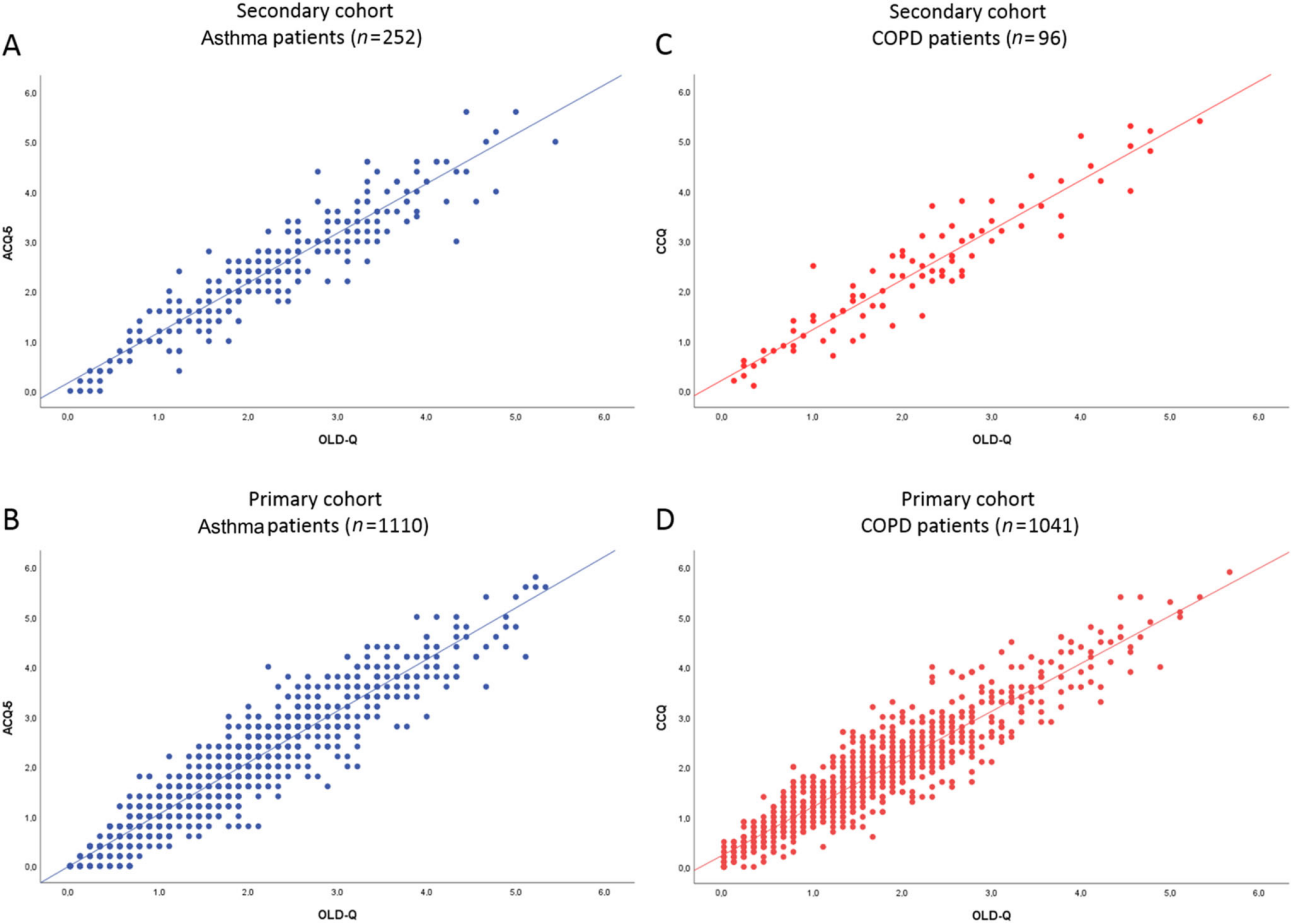
Correlation plots. Correlation plot showing the correlation between the ACQ and the OLD-Q in asthma patients in secondary cohort (**A**) and primary cohort (**B**) and the correlation between the CCQ and the OLD-Q in COPD patients in secondary cohort (**C**) and primary cohort (**D**). The Pearson correlation coefficient (*R-score*) was respectively 0.93 (**A**), 0.94 (**B**), 0.94 (**C**), and 0.93 (**D**).

In asthma patients, the correlation coefficient of the ACQ and total score of OLD-Q was 0.93 in the secondary cohort and 0.94 in the primary cohort. The correlation coefficient between the CCQ and the OLD-Q in COPD patients was 0.94 in the secondary cohort and 0.93 in the primary cohort, Fig. 4. The correlation coefficient in COPD (*n* = 61) between CAT and OLD-Q was 0.723 and between CCQ and CAT was 0.731. The correlation coefficient in asthma (*n* = 197) between AQLQ and OLD-Q was -0.686 and for AQLQ and ACQ total -0.652. The Cronbach’s alpha of the OLD-Q in the secondary care was for asthma: 0.877, for COPD patients: 0.885, and for ACO patients: 0.884. In primary care, the Crohnbach’s alpha was for asthma patients 0.867, for COPD 0.858 and for ACO 0.858.

## Discussion

In this study, we searched for statistical and clinical overlap in questions contained in the asthma control questionnaire and the clinical COPD questionnaire in asthma, COPD and ACO in a primary and secondary cohort. This new approach could increase the efficacy of the assessment of the disease burden in asthma and COPD by merging the ACQ and CCQ based on statistical correlations. Our study explored the possibility of creating a new questionnaire and showed that a combination of nine questions of the ACQ and CCQ may be sufficient to assess disease burden in obstructive lung disease. This 9-item Obstructive Lung Disease Questionnaire, was strongly correlated to respectively the ACQ in patients with asthma and the CCQ in patients with COPD.

There has been an increasing emphasis on the importance of distinguishing between asthma and COPD. Despite their similar clinical features, the treatment approaches for asthma and COPD differ significantly. This is particularly crucial when considering treatment with ICS medication. In asthma, there’s a need to avoid under-treatment by withholding ICS, whereas in COPD, there’s a risk of over-treatment with ICS^21^. Therefore, it’s essential to emphasize that the OLD-Q, along with the ACQ and CCQ, is developed as a tool to assess disease burden rather than as a diagnostic tool. These questionnaires should not replace the necessary steps and tests required for accurate diagnosis. Given that the OLD-Q is applicable to all patients with obstructive lung disease, it remains valuable throughout both the diagnostic and follow-up phases.

During the selection procedure of the questions, in addition to selecting one question from each component, various other factors were also taken into consideration. In the development cohort, the ACQ-2 was correlated to the CCQ-3 and CCQ-4 in component 3. Initially, this question about dyspnoea in the morning seems clinically unrelated to questions about feeling concerned or depressed. However, a systematic review showed that the burden of COPD is more severe in the morning and that these symptoms are associated with a lower quality of life^22^. Nevertheless, the components of the three reproduction cohorts did not contain the ACQ 2. Therefore, the ACQ-2 was not merged with the CCQ-3 and CCQ-4. In total, six questions were not related to any of the components in the primary or secondary cohort. These questions were included in our selection, because removing them would result in loss of information. Moreover, the CCQ and ACQ were originally formulated by expert opinions for COPD and asthma separately. The intention of this research was not to remove questions, but to merge questions with statistical overlap to be useful for patients with asthma, COPD and asthma-COPD overlap.

To our knowledge, this study is the first to attempt to integrate and reduce the ACQ and CCQ to develop a pragmatic set of questions to assess disease burden in obstructive lung disease in daily practice. Modifying questionnaires to extend their applicability to a broader patient group is feasible, as shown in a recent study that transformed the COPD Assessment Test (CAT) into the Chronic Airways Assessment Test (CAAT). The CAAT seems applicable in both COPD and asthma^23^. Also, the Assessment of Burden of Chronic Obstructive Pulmonary Disease (ABC)-scale was created as an adaptation of the CCQ by adding extra domains^24^. Later, the ABC-tool was extended to the Assessment of Burden of Chronic Conditions (ABCC)-tool, adding information on comorbidity^25^. The ABC- and ABCC-tool were created for another purpose than the OLD-Q. Whereas the OLD-Q is created to monitor disease burden in daily practice, the ABC- and ABCC-tool are used for an in-depth assessment of a patient to help healthcare-professionals to formulate a personalized treatment plan together with their patient^24,25^. Some alternative questionnaires exist for evaluating the quality of life or disease burden in respiratory disease. The Quality-of-life for Respiratory Illness Questionnaire (QoL-RIQ), for instance, is a questionnaire for assessment of disease burden and is validated in both asthma and COPD. However, this questionnaire is not practical due to the large number of questions^26^. For that reason, the reduced ten-item version RIQMON-10 was developed^27^. The Respiratory Symptoms Questionnaire (RSQ) is another questionnaire for assessment of respiratory symptoms regardless of a specific diagnosis. Similar to the OLD-Q, the RSQ was developed as a practical four-item tool. However, the development of the RSQ was based on the GINA and GOLD guidelines^9,10^, whereas our selection of questions is based on data and statistical analysis of two commonly used questionnaires^28^. The RIQMON-10 and RSQ are not commonly used in daily care. Whereas the ACQ and CCQ are frequently used, we expect that the selection of questions in the OLD-Q are familiar to both healthcare professionals and patients, so it should be more easily adopted into daily practice. Nevertheless, it’s important to mention that the ACQ and CCQ aren’t universally used across all countries. In some regions, the CAT or ACT are more prevalent. Consequently, adapting to this new questionnaire might require additional time in such countries.

Our study has a number of significant strengths and some limitations. The first strength of the study is that the ACQ and CCQ were collected on the same day in a well-defined primary and secondary cohort. The second strength is the methodology: we developed the OLD-Q by merging two well-known questionnaires for two diseases that show clinical and physiological overlap. The ACQ and CCQ were developed based on expert opinion, validated, and are used in primary, secondary and tertiary care. Furthermore, the ACQ and CCQ are self-administered, so health care professionals do not influence the results. We examined the relationship within the group of questions by PCA and consequently reduced questions. This statistical analysis can visualize correlations between questions in large numbers of patients, which is impossible for a clinician to observe. By developing the questionnaire in a secondary care cohort and reproducing the results in a primary cohort, we assume that this new questionnaire could be used in primary and secondary care. Third, the OLD-Q showed a very strong correlation with the ACQ in asthma patients and in CCQ in patients with COPD. Fourth and final, with a high value of Cronbach’s alpha, we showed that the scale of the outcome is reliable with internal consistency.

This study has some limitations. First, the COPD group was relatively small in the secondary cohort (*n* = 92). Second, the PCA of the secondary cohort with ACO patients showed some other results compared to the development cohort and to the other three reproduction cohorts, most likely because this patient group was small compared to the other patients group. Therefore, these results were be interpreted with caution. Third, we investigated the correlation between the OLD-Q and the original CCQ and ACQ as a gold standard, either for patients with COPD or asthma. The ACQ covers a greater number of aspects related to disease control, while the CCQ places a stronger focus on quality of life. One could argue that we should also validate the OLD-Q with another disease burden questionnaire, such as the St. George Respiratory Questionnaire (SGRQ), which is validated for both patients with asthma and COPD. However, significant differences are unlikely since our results show a similar correlation between the OLD-Q and the AQLQ in asthma, or the CAT in COPD, to the correlations previously documented in the literature between the ACQ and AQLQ or CCQ and CAT^29^. Fourth, the PCA showed that it is statistically possible to reduce the number of questions without interfering with the total score. This reduction may have resulted in the loss of clinically relevant information, for example to differentiate in exercise ability. However, this selection of questions is developed for a particular purpose: that is monitoring disease burden in daily care, and not as an in-depth assessment of the patient.

In this study, we established the possibility of developing a new questionnaire by using the ACQ and CCQ. The next steps include testing the OLD-Q for convergent and divergent validity, differential and linearity of item response, item response characteristics and cognitive debriefing^5,6^. Furthermore, in this study the questions used in the OLD-Q have been presented in the context of the ACQ and CCQ, so previous questions may have influenced the responses to subsequent questions. To further validate the OLD-Q as a new questionnaire, the questions should be presented in the correct order. This will help ensure that responses are not biased by previous questions and that the questionnaire is reliable and valid. A prospective study in a real-world setting with patients with asthma, COPD and ACO is warranted to validate the OLD-Q with the SGQR at two different time points to confirm validity and investigate the test-retest reliability of this pragmatic set of questions.

In conclusion, this potential new practical disease burden questionnaire is clinically relevant considering the similar outcomes for both primary care and secondary care. These results indicate that the OLD-Q could serve as a tool for assessing disease burden starting at the time of diagnosis and continuing through the follow-up period. In this way patients need to answer fewer questions than in the current situation, which is time-efficient for both patients and health professionals.

### Supplementary information

Appendix

Reporting Summary

## Data Availability

All data relevant to the study are included in the article or uploaded as supplementary information. The datasets used and/or analyzed during the current study are available from the corresponding author upon reasonable request.
