# Supplementary material for: Measuring burden of disease in both asthma and COPD by merging the ACQ and CCQ: less is more?
Source: NPJ Prim Care Respir Med. 2024 May 3;34:8. doi: 10.1038/s41533-024-00364-z (PMC11068875; doi:10.1038/s41533-024-00364-z)
Supplement: Supplementary file 1 — Appendix [file 41533_2024_364_MOESM1_ESM.pdf]

Appendix

Suppl. Figure 1a and 1b Median ACQ-scores and CCQ-scores with IQR in development cohort (asthma and COPD patients in secondary data base)

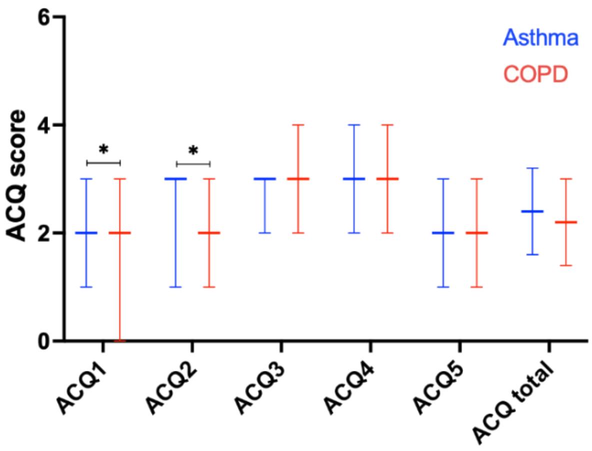

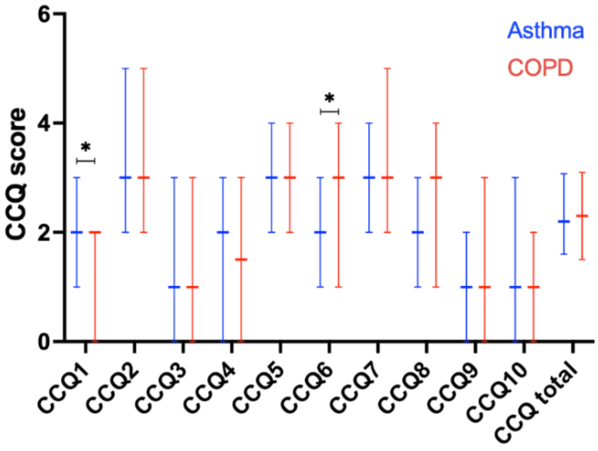

*Statistically significant difference between asthma and COPD patients in ACQ1 (p-value 0,019), ACQ2 (p-value 0,011), CCQ1 (p-value 0,002) and CCQ6 (p-value 0,01) scores, calculated by Mann-Whitney U test

Suppl. Table 1. Patient Characteristics of Asthma COPD overlap patient group in secondary and primary care

**Characteristics** *Asthma-COPD Overlap*	**Secondary care cohort (n=53)**	**Primary care cohort (n=355)**
**Female sex, n(%)**	22 (42)	165 (47)
**Age, median [IQR]**	61 [55-68]	59 [50-68]
**BMI, median [IQR]**	26 [24-30]	27 [24-31]
**Active smokers, n(%)**	19 (36)	148 (42)
**FEV-1 pre percentage predicted, median [IQR]**	62 [51-72]	65 [53-76]
**FEV-1 post percentage predicted, median [IQR]**	69 [58-79]	74 [61-85]
**FEV-1/FVC, median [IQR]**	55 [44-63]	61 [54-66]

Suppl. Table 1. Patient Characteristics of Asthma COPD overlap patient group in secondary and primary care

Suppl Table 2. Questions of the ACQ and CCQ that were not included in the OLD-Q

ACQ 3<br><br>In general, during the past week, how limited were you in your activities because of your asthma?	0 Not limited at all<br>0 Very slightly limited<br>1 Slightly limited<br>2 Moderately limited<br>3 Very limited<br>4 Extremely limited<br>6 Totally limited
CCQ 2<br><br>On average, during the past week,<br>how often did you feel short of breath doing physical activities?	0. Never<br>1. Hardly ever<br>2. A few times<br>3. Several times<br>4. Many times<br>5. A great many times<br>6. Almost all the time
CCQ 4<br><br>On average, during the past week,<br>how often did you feel depressed (down) because of<br>your breathing problems?	0. Never<br>1. Hardly ever<br>2. A few times<br>3. Several times<br>4. Many times<br>5. A great many times<br>6. Almost all the time
CCQ 5<br><br>In general, during the past week,<br>how much of the time did you cough?	0. Never<br>1. Hardly ever<br>2. A few times<br>3. Several times<br>4. Many times<br>5. A great many times<br>6. Almost all the time
CCQ 8<br><br>On average, during the past week, how limited were you in these activities because of your breathing problems: moderate physical activities?	0. Not limited at all<br>1. Very slightly limited<br>2. Slightly limited<br>3. Moderately limited<br>4. Very limited<br>5. Extremely limited<br>6. Totally limited/ or unable to do
CCQ 9<br><br>On average, during the past week, how limited were you<br>in these activities because of your breathing problems:<br>daily activities at home (such as dressing, washing yourself)?	0. Not limited at all<br>1. Very slightly limited<br>2. Slightly limited<br>3. Moderately limited<br>4. Very limited<br>5. Extremely limited<br>6. Totally limited/ or unable to do
